# Zika virus infection modulates the bacterial diversity associated with *Aedes aegypti* as revealed by metagenomic analysis

**DOI:** 10.1371/journal.pone.0190352

**Published:** 2018-01-02

**Authors:** Luis E. M. Villegas, Thais B. Campolina, Nilton R. Barnabe, Alessandra S. Orfano, Barbara A. Chaves, Douglas E. Norris, Paulo F. P. Pimenta, Nagila F. C Secundino

**Affiliations:** 1 Laboratory of Medical Entomology, René Rachou Research Centre–FIOCRUZ, Minas Gerais, Brazil; 2 Tropical Medicine Foundation Dr. Heitor Vieira Dourado, Manaus, Amazonas, Brazil; 3 Department of Molecular Microbiology and Immunology, Johns Hopkins Bloomberg School of Public Health, Baltimore, Maryland, United States of America; Universidade Federal do Rio de Janeiro, BRAZIL

## Abstract

Zika is a re-emerging infection that has been considered a major threat to global public health. Currently at least 100 countries are at risk of Zika virus (ZIKV) transmission. *Aedes aegypti* is the main mosquito vector in the Americas. This vector is exposed to, and interacts symbiotically with a variety of microorganisms in its environment, which may result in the formation of a lifetime association. Here, the unknown effect that ZIKV exerts on the dynamic bacterial community harbored by this mosquito vector was investigated using a metagenomic analysis of its microbiota. Groups of *Ae*. *aegypti* were experimentally fed on sugar, blood and blood mixed with ZIKV, and held for 3 to 7 days after blood meal and eggs development respectively. The infected groups were processed by qPCR to confirm the presence of ZIKV. All groups were analyzed by metagenomics (Illumina Hiseq Sequencing) and 16S rRNA amplicon sequences were obtained to create bacterial taxonomic profiles. A core microbiota and exclusive bacterial taxa were identified that incorporate 50.5% of the predicted reads from the dataset, with 40 Gram-negative and 9 Gram-positive families. To address how ZIKV invasion may disturb the ecological balance of the *Ae*. *aegypti* microbiota, a CCA analysis coupled with an explanatory matrix was performed to support the biological interpretation of shifts in bacterial signatures. Two f-OTUs appeared as potential biomarkers of ZIKV infection: *Rhodobacteraceae* and *Desulfuromonadaceae*. Coincidentally, both f-OTUs were exclusively present in the ZIKV- infected blood-fed and ZIKV- infected gravid groups. In conclusion, this study shows that bacterial symbionts act as biomarkers of the insect physiological states and how they respond as a community when ZIKV invades *Ae*. *aegypti*. Basic knowledge of local haematophagous vectors and their associated microbiota is relevant when addressing transmission of vector-borne infectious diseases in their regional surroundings.

## Introduction

Zika is a re-emerging disease, with a recent outbreak in Brazil in 2015 that resulted in Zika being considered a major threat to global public health. Currently at least 100 countries are at risk of Zika virus (ZIKV) transmission (http://www.cdc.gov/zika/geo/active-countries.html). *Aedes aegypti*, the main mosquito vector in the Americas, has worldwide distribution across tropical and subtropical regions resulting in almost half of the world’s population at a risk of infection [1]. Transmission of ZIKV as well as other arboviruses, such as the Chikungunya and dengue viruses by mosquitoes in Brazil occurs steadily throughout the year in most regions, favoring their rapid dissemination [2].

Insects are exposed to, and interact symbiotically with a variety of microorganisms [3], some of which they acquire from their ecosystems and through feeding resulting in an association that may remain throughout their life spans [4,5]. Several key members of the native microbiota are transstadially transmitted during multiple life stages of their host [5,6]. To date, bacteria are most studied component of the microbial community in mosquito models [7–9]. This community may fluctuate in both richness and abundance during the developmental and adult phases of the mosquito [10, 11], with some bacterial OTUs (operational taxonomic units) that remain associated but dynamically shift towards a high abundance in response to physiological conditions [11–13]. In addition, studies have shown that disrupting the homeostasis of microbiota, for example, through antibiotic therapy, affects susceptibility towards pathogens, including the models *Anopheles gambiae* and *Ae*. *aegypti* when exposed to *Plasmodium falciparum* and dengue virus, respectively [14–16]. Although the metabolic or physiological role that autochthonous bacteria play within arthropod vectors, as well as their potential interactions with human pathogens are not fully understood, bacteria may directly or indirectly modulate vector competence, and have an impact on vector bionomics [8,14–18].

The association between microbial consortia and arthropod hosts has been described for a large variety of vector models [8,19]. Herein, we explored the potential effect that ZIKV exerts on the dynamic bacterial community harbored by the main mosquito vector *Ae*. *aegypti*. For this purpose, we performed the metagenomic taxonomic profiling of the Brazilian *Ae*. *aegypti* mosquito under distinct physiological conditions, including ZIKV infection. The goal was to determine whether ZIKV infection causes a noticeable ecological disruption of the profile of bacterial abundance in the holobiont [20]. To address this, we performed a constrained comparison of community structure using β-diversity as a measure of the variance within a community data matrix [21,22]. The multivariate analyses revealed how the bacterial community profile not only shifts across the life stages of *Ae*. *aegypti*, but also responds to the presence of invading ZIKV or indicates the condition of the mosquito, which suggests bacterial f-OTUs as potential ecological markers of infection within the mosquito vector under the experimental conditions tested.

## Materials and methods

### *Aedes aegypti* and viral infection

Experimental infection was conducted using 3–5 day-old female *Ae*. *aegypti* mosquitoes. A well-established Brazilian closed colony of *Ae*. *aegypti* (strain PP-Campos), maintained at the Laboratory of Medical Entomology, Fiocruz-MG, Brazil, was used in this study. Mosquito eggs were collected using ovitraps in the city of Campos dos Goitacazes, State of Rio de Janeiro, Brazil, in 2000. Mosquitoes were reared and maintained under standard insectary conditions (27°C, 80% relative humidity, 16-hour light/8-hour dark photoperiod).

The mosquitoes were infected via a membrane-feeding assay using a glass-feeding device that was filled with mouse blood containing a ZIKV isolate (ZIKV-SPH, ZIKV/*H*. *sapiens*/Brazil/SPH/2015) that is currently in circulation in Brazil [23]. The mosquitoes were allowed to feed on blood infected with ZIKV at a titer of 1 x 10^5^ PFU for 1 hour. Following blood feeding, approximately 100 fully engorged *Ae*. *aegypti* mosquitoes were obtained to form each group and biological replicates: a) sugar-fed (fed *ad libitum* with sugar), (b) blood-fed (normal non-infected blood), and (c) ZIKV-infected blood-fed. After blood digestion, two groups were re-manned as (d) gravid (7 days post non-infectious feeding (group b) with developing eggs), and (e) ZIKV-infected gravid (7 days post ZIKV infectious feeding (group c) with developing eggs). All mosquitoes were maintained with *ad libitum* access to 10% glucose solution until the end of experiment. The gravid groups allowed determination of whether the ZIKV infection had an effect on the bacterial profile of *Ae*. *aegypti* mosquitoes during egg development. For the ZIKV-infected blood-fed and ZIKV-infected gravid groups, the presence of the virus was verified by TaqMan real-time qPCR (quantitative real time PCR) assay, as described [24] with 100% of the mosquitoes infected at the 3^rd^ and 7^th^ day after the infectious blood meal with a median of 10^6^ and 10^9^ cDNA copies/ mosquito, respectively).

### DNA extraction

Pooled samples consisted of 30 *Ae*. *aegypti* mosquitoes from each experimental group (three biological independent replicates composed by 10 mosquitoes). Before genomic DNA extraction, each individual mosquito in the pooled sample was surface-sterilized by washing with distilled sterile water, and then submerged for 10 s in 70% ethanol, one min in 1% hypochlorite, and one min in PBS (phosphate-buffered saline) [25]; the washing steps were performed three times. This procedure is routinely used for sterilization of insect surfaces without considerable impact on the profiles of internal bacterial communities [26]. Genomic DNA was then extracted using the DNeasy Blood and Tissue Kit (Qiagen, Hilden, Germany) following the manufacturer’s instructions.

### Amplicon-oriented metagenomic profiling of bacterial communities

Next Generation Sequencing (NGS) of the samples using the Illumina MiSeq platform and targeting the V3–V4 hypervariable region of the 16S rRNA gene was outsourced to Macrogen (https://dna.macrogen.com/eng/), and 300-bp-long paired-end reads were obtained.

All the data were processed in accordance with standard bioinformatics pipelines, which accounted for the following: Quality Control, clustering of operational taxonomic units (OTUs;) clustering (CD-HIT-OTUT at an identity threshold of 97%), and analysis of community richness analysis (Mothur v1.35.1) using taxonomic assignments based on the release 128 of the SILVA rRNA and in-house software or algorithms.

### Analyses of the composition of the bacterial communities

The diversity richness and abundance of the bacterial component of the microbiota of each experimental *Ae*. *aegypti* group was studied in terms of α-diversity, presence and absence patterns, and β-diversity-based multivariate analyses on a relative abundance matrix. When conventional methods of α-diversity analysis were considered, we opted to report a measure of community evenness (the Shannon diversity index) to address the disturbance that ZIKV replication might have caused to the homeostasis of the bacterial community. This parameter of community structure, in particular, has been linked to the biological phenomenon of successful pathogen invasion [27]. In order to visualize the presence and absence patterns of the f-OTUs per experimental group, circular plots were generated from the tabular indices using Circos online table viewer v0.63–9 [28]. By cross-referencing the indices, sets of exclusive f-OTUs per group, as well as a core set of taxa that were present in all of the experimental *Ae*. *aegypti* groups were revealed. When the variance among the groups (β-diversity) in terms of the profiles of OTU abundance was explored, the criteria tested and described by Audsley et al [29] were applied, using a cut-off of 10 HQ reads at the taxonomic level of family (f-OTUs) to build the data matrix for downstream ordination analysis.

The microbial community composition comparisons were based on pairwise dissimilarity measurements and were performed on a matrix encompassing the relative abundance profiles of the bacterial OTUs from each tested group. For a more conservative approach, analyses were based at the family level to encompass higher OTU counts and perform a more robust explorative analysis and constrained ordination, by reducing the number of zeroes in the data matrix. A variance- stabilizing transformation was applied to the relative abundance matrix to reduce dispersion, expressing the values as the arcsin√x [30, 31].

The multivariate analyses were performed on the transformed dataset of abundance profiles considering suggested parameters of microbial ecology, based on the Bray-Curtis (B-C) index in order to exclude joint absence [22,30,32]. As described in recent literature for metagenomic studies in vector biology [33,34], the conventional approach has been used for weighted and/or unweighted UniFrac distances [35, 36]. This may be a frequently used approach since it is implemented in the widely used pipelines for the analysis of microbial community sequence, QIIME [37] and Mothur [38]. Although this is a currently popular and highly informative dissimilarity measure to compute pairwise distances between microbial communities, the remarks of the developers regarding when to use the dissimilarity measure for studies on β-diversity and community composition should be considered. Since our study focused on individuals from the same population, which were reared and experimented upon under homogeneous conditions, dissimilarities were estimated using the B-C index. In this context, our hypothesis of the modulation of community structure by the pathogen (ZIKV) did not require consideration of the phylogenetic relationships among the bacterial OTUs, or their evolutionary history when the pairwise comparisons between the elements of the data matrix were performed.

As Kuczynski et al [39] reported, the use of phylogenetic-based measures requires substantial additional expertise to interpret the effect of the phylogenetic parameters on the ordination methods and their ecological interpretation. Unless the evaluation of the host-microbiota phylosymbiosis [40, 41], or the evaluation of geographic and niche-oriented effects [34] are within the scope of the study, performing other distance calculations as well is suggested in order to approach a situation wherein bacterial markers are used to compare experimental groups.

The spatial distribution patterns of both objects and response variables in a multidimensional ordination space were analyzed for identifying underlying explanatory variables that influenced their variances. A Detrended Canonical Correspondence Analysis (DCCA) was performed to determine whether a linear or unimodal ordination method could be applied to the data. The longest gradient obtained was larger than 3.0 SD units, which indicated that a unimodal model-based constrained method was suitable [30, 32]. Therefore, a Canonical Correspondence Analysis (CCA, scaling 2) was performed using an additional explanatory matrix with “dummy” binary variables referring to the following conditions: presence of blood in the midgut, ZIKV infection, and a state of gravidity. By constraining the multivariate analysis to these explanatory variables, the potential ecological preference of the f-OTUs towards conditions that could influence their abundance or thriving in a particular niche state was explored [30,42]. Thus, the CCA triplot permitted us to observe how the explanatory variables (vector arrows) influenced the community variance, which was represented in the constraining axis. In addition, by also selecting the scaling 2 parameter scaling it was possible to observe the position of the maximum values of key response variables along an explanatory variable by means of the orthogonal distance between the f-OTU points and the arrow vectors. All of the abovementioned analyses were performed using the Vegan package of Rstudio version 0.98.507 [43].

### Ethics statement

This study was conducted in accordance with the recommendation in the Manual for the Use of Animals (FIOCRUZ, Ministry of Health of Brazil, National decree, Nr. 3179). The protocol was approved by the Ethics Committee for the Use of Animals of Fundação Oswaldo Cruz (FIOCRUZ, Ministry of Health of Brazil, Nr. L-1715).

## Results and discussion

### Taxonomic profiling of the bacterial community

NGS generated a total of 123,973 valid HQ reads that were identified as bacteria in the *Ae*. *aegypti* mosquitoes and which were distributed among the following experimental groups: 21,383 in the sugar-fed group, 27,235 in the blood-fed group, 24,072 in the ZIKV-infected blood-fed group, 24,594 in the gravid group and 25,649 in the ZIKV-infected gravid group. These reads corresponded to Ninety seven f-OTUs that fell within the following 14 phyla (f-OTU number within parentheses): *Acidobacteria* (1), *Actinobacteria* (10), *Bacteroidetes* (12), *Chlamydiae* (3), Cyanobacteria/Chloroplast (1), *Deinococcus-Thermus* (1), *Firmicutes* (16), *Gemmatimonadetes* (1), *Ignavibacteriae* (1), Nitrospirae (1), *Planctomycetes* (2), *Proteobacteria* (41), *Spirochaetes* (3), and *Verrucomicrobia* (4). The presence or absence patterns of the f-OTUs within each experimental group are depicted in S1 Fig. As Kuczinski et al [39] thoroughly discussed, the depth of sequencing does not determines the capacity to reveal underlying biological patterns from datasets of community structures. Here, working downstream with read counts allowed for the construction of a conservative and robust data matrix, as it has been shown that by choosing appropriate ordination methods and dissimilarity measures as few as 100 sequences can reveal relevant ecological patterns within the structure of a bacterial community structure [44].

### Community evenness

When community composition was explored in terms of α-diversity, the reported evenness indices for the experimental *Ae*. *aegypti* groups were as follows: 8.00 for the sugar-fed group, 8.43 for the blood-fed group, 5.54 for the ZIKV-infected blood-fed, 8.11 for the gravid group, and 6.37 for the ZIKV-infected gravid group. Therefore, when community richness was addressed in terms of evenness, the Shannon diversity indices revealed that there were more bacterial OTUs, with the total reads more equitably distributed, in the noninfected adult *Ae*. *aegypti* mosquitoes. In contrast, the read abundance was less homogenously distributed amongst the bacterial taxa in the community when exposed to ZIKV. This trend was similarly observed on the native microbiota of *Lutzomyia longipalpis* when exposed to the parasite *Leishmania infantum chagasi* [33]. Together, these data suggest that successful invasion and concomitant transmission of these two vector-borne pathogens is reflected by the ecological disturbance of the native bacterial community in the host.

### Overview of community composition, and identification of exclusive and core microbiota per group

**Fig 1:**
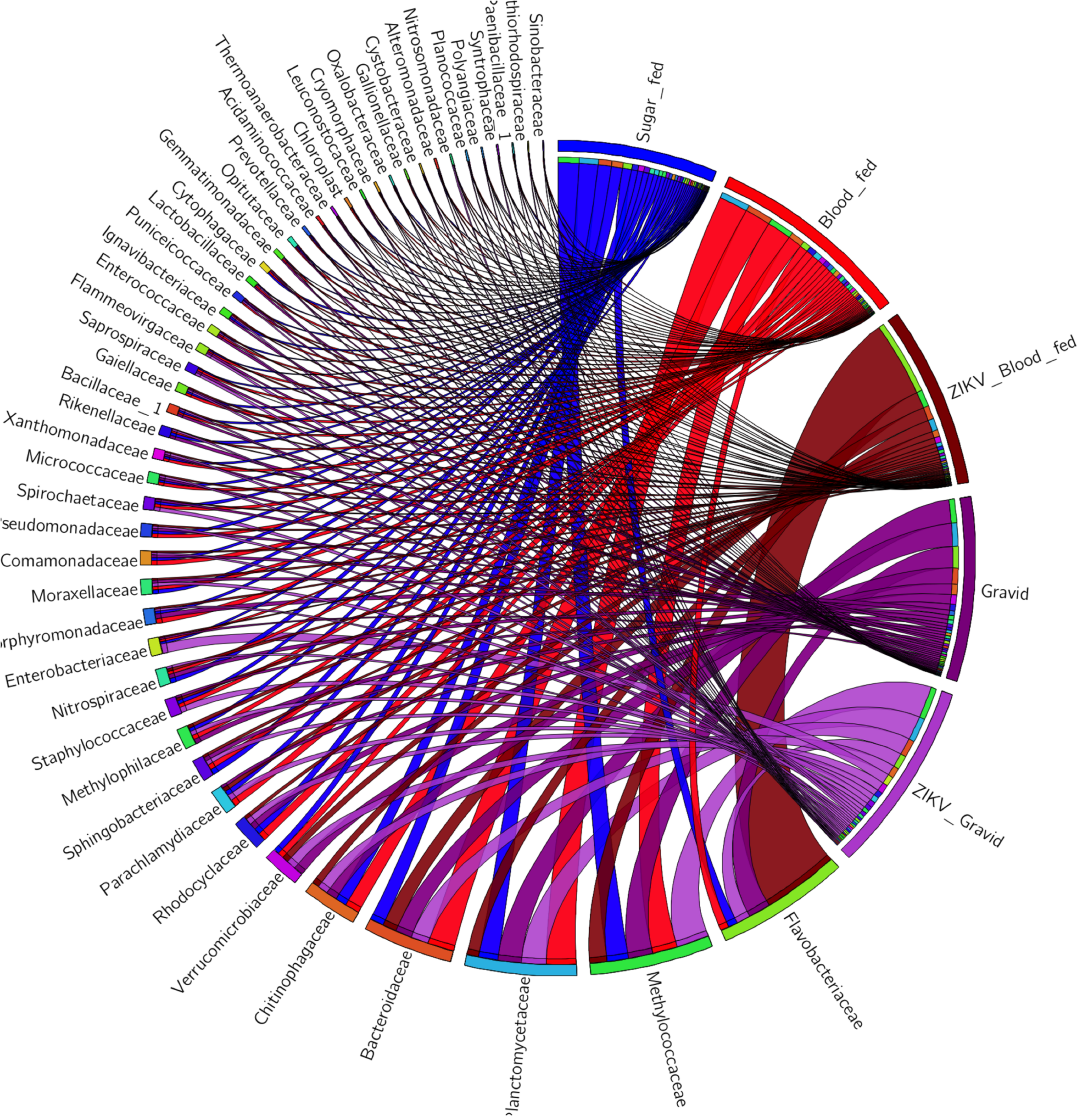
Relative abundance of the core bacterial taxa associated with laboratory-reared adult *Ae*. *aegypti* mosquitoes. The circular plot depicts the abundance of the core bacterial taxa identified by crossing the indices of the f-OTUs per group for the experimental *Ae*. *aegypti* groups: sugar-fed (blue); blood-fed (red); ZIKV-infected blood-fed (maroon); Gravid (purple); and ZIKV-infected gravid (lilac). The bacterial taxa and experimental groups are indicated next to their ribbons on the perimeter of the circle. The width of the ribbon and the position of the f-OTUs from the bottom to the top (on each segment) reflect the abundance of the bacterial taxa and their presence within each group.

When the index of f-OTUs of each experimental *Ae*. *aegypti* group was compared (S1 Table), group specific and core and bacterial taxa were identified, as depicted by the circular plots (Fig 1). The subsets were identified by considering the criterion of being present in all groups or absent in at least one of them. It was inferred that 49 OTUs remained associated with the *Ae*. *aegypti* mosquitoes under all the tested experimental conditions. This core assembly encompassed 50.5% of the predicted reads from the dataset, with 40 Gram-negative and 9 Gram-positive bacterial families. These results suggested that although habitat, food source, and life stage may modulate the richness profile, some bacterial OTUs within this broad taxonomic category could thrive under changing conditions of the holobiont [5,8,10,11], and may be essential to the host as a systemic symbiotic core [4,40,45].

Our data differed from that of a recent study using uninfected *Ae*. *aegypti* from a field and a colony, which revealed a core microbiota assembly of 19 bacterial taxa, including 10 f-OTUs [11] Particularly, the differences observed in terms of OTU richness could be due to the NGS platform used and to the targeted region(s) of the 16S rRNA gene, as they each possessed different taxonomic resolution [46]. Such core assemblies of host-associated bacterial OTUs have been described in mosquitoes including anophelines, and were determined by different criteria, such as tissue specificity [46,47], life stage [10,11,13], or geographic distribution, as suggested by Villegas and Pimenta [9].

**Fig 2:**
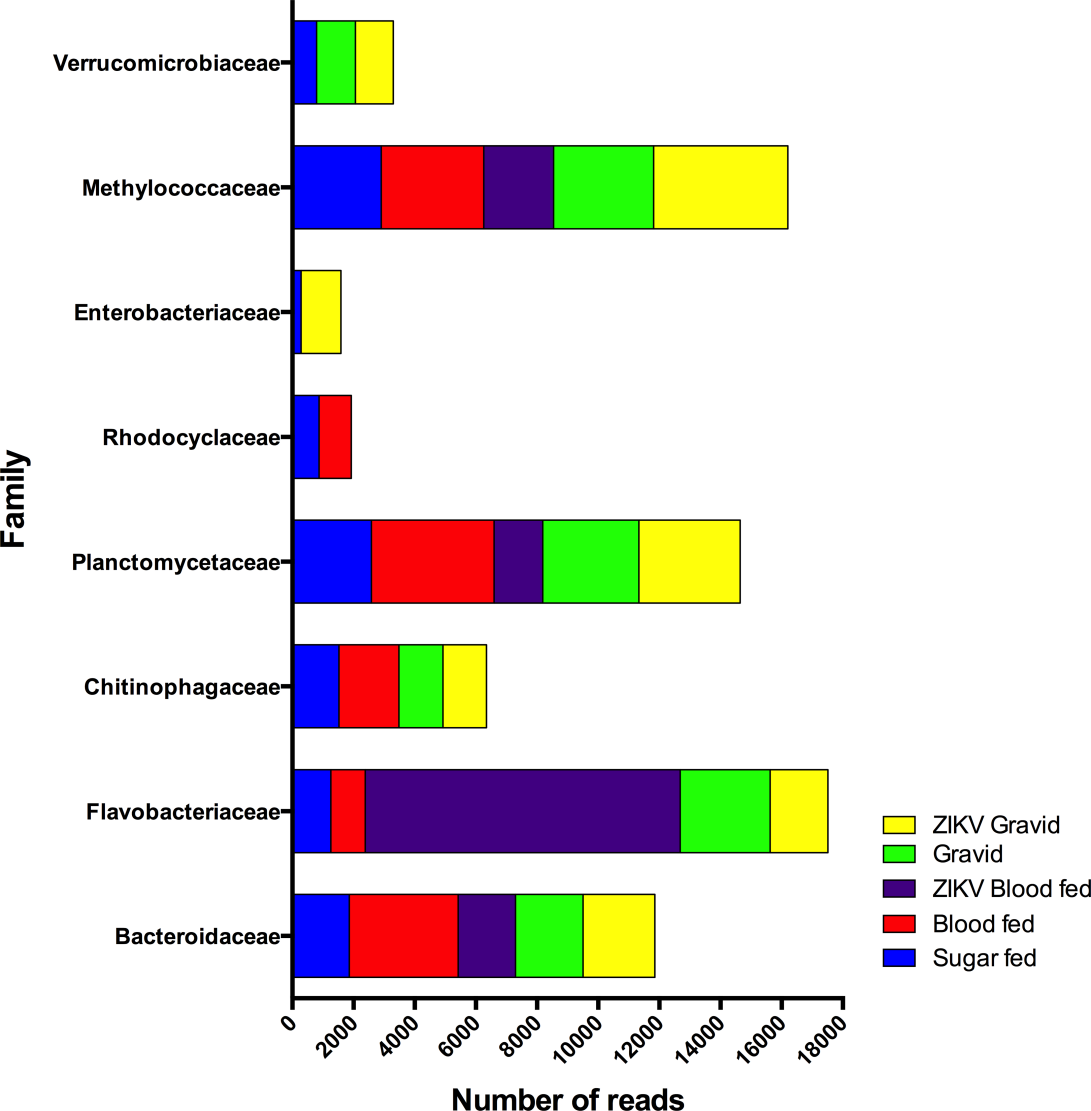
Raw abundance of the core bacterial communities per *Ae*. *aegypti* group. The presence of arbitrarily selected core f-OTUs per group is shown as stacked bars to explore the dynamic shifts exhibited within each experimental group, which is represented by a color as indicated in the figure.

Fluctuations in abundance within the core group of taxa were observed under different physiological conditions and revealed a dynamic community of bacteria (Figs 1 and 2). In the ZIKV blood-fed group the Flavobacteriacae family was the furthermost prevalent, accounting for 60% of bacterial OTU reads, decreasing in abundance in the other mosquito groups in the following order: Gravid (20%) > ZIKV Gravid (10%) > Sugar-fed (6%) > and blood-fed (4%). The initial induction of *Flavobacteria* by ZIKV appears to be important in the first days of infection but diminish after 7 days as is observed in the mosquitoes of the group ZIKV Gravid. The thriving abundance of this f-OTU in the infected blood-fed mosquitoes (ZIKV-infected blood-fed group) was in contrast with previous studies that reported its high relative abundance in laboratory-reared sugar-fed *Ae*. *aegypti* mosquitoes (represented at the genus level by *Elizabethkingia* [11]) and blood-fed *An*. *Gambiae* collected from the field and reared in a microcosmos [10]. This contrasting relative abundance could be attributable to physiological differences between strains and genera, or sequencing (effects of the primers and platform) and downstream computational processing (e.g. taxonomic assignment and reference database selection) [46,48–50].

The f-OTUs *Bacteroidaceae* and *Planctomitaceae* exhibited a similar abundance pattern when they were present in all the experimental *Ae*. *aegypti* groups. *Methylococcaceae* was the second most abundant family across the experimental groups, and exhibited a relatively homogeneous bacterial community genera prevalence in the uninfected groups, while being the dominant f-OTU in the ZIKV infected groups. Surprisingly, *Enterobacteriaceae* was not the most responsive in terms of biomass proliferation (measured in terms of read abundance) in the blood-fed group, contrary to what was reported in anophelines [10,12]. It has been suggested that *Enterobacteriaceae* would potentially dominate and thrive under these conditions because of its redox potential [10,51], however this f-OTU was predominant in the ZIKV-infected gravid group.

To further show these fluctuations among the most abundant taxa, the f-OTUs encompassing less than 1000 valid reads were arbitrarily excluded, which revealed a remaining set of 8 families (Fig 2). The influence of blood feeding on the diversity profile of these 8 families present in the experimental sugar-fed group was analyzed. Conditions of oxidative stress and high protein content due to blood-digestion may favor *Methylococcaceae*, *Rhodocyclaceae*, *Planctomycetaceae*, *Chitinophagaceae*, *Flavobacteriaceae*, and *Bacteroidaceae* in order to outcompete *Enterobacteriaceae* and *Verrucomicrobia* [10,51]. However, in the presence of ZIKV, *Flavobacteriaceae* would be the thriving taxon in a community with lower richness as indicated by the Shannon diversity indices. As digestion occurred, the gravid *Ae*. *aegypti* group would then harbor a community wherein *Rhodocyclaceae* and *Enterobacteriaceae* would be less abundant, with *Flavobacteriaceae*, *Planctomycetaceae* and *Methylococcaceae* being the dominant community members. In the presence of ZIKV and during vitellogenesis, the latter taxa dominated the community, and *Enterobacteriaceae* was again detected above the threshold set.

**Fig 3:**
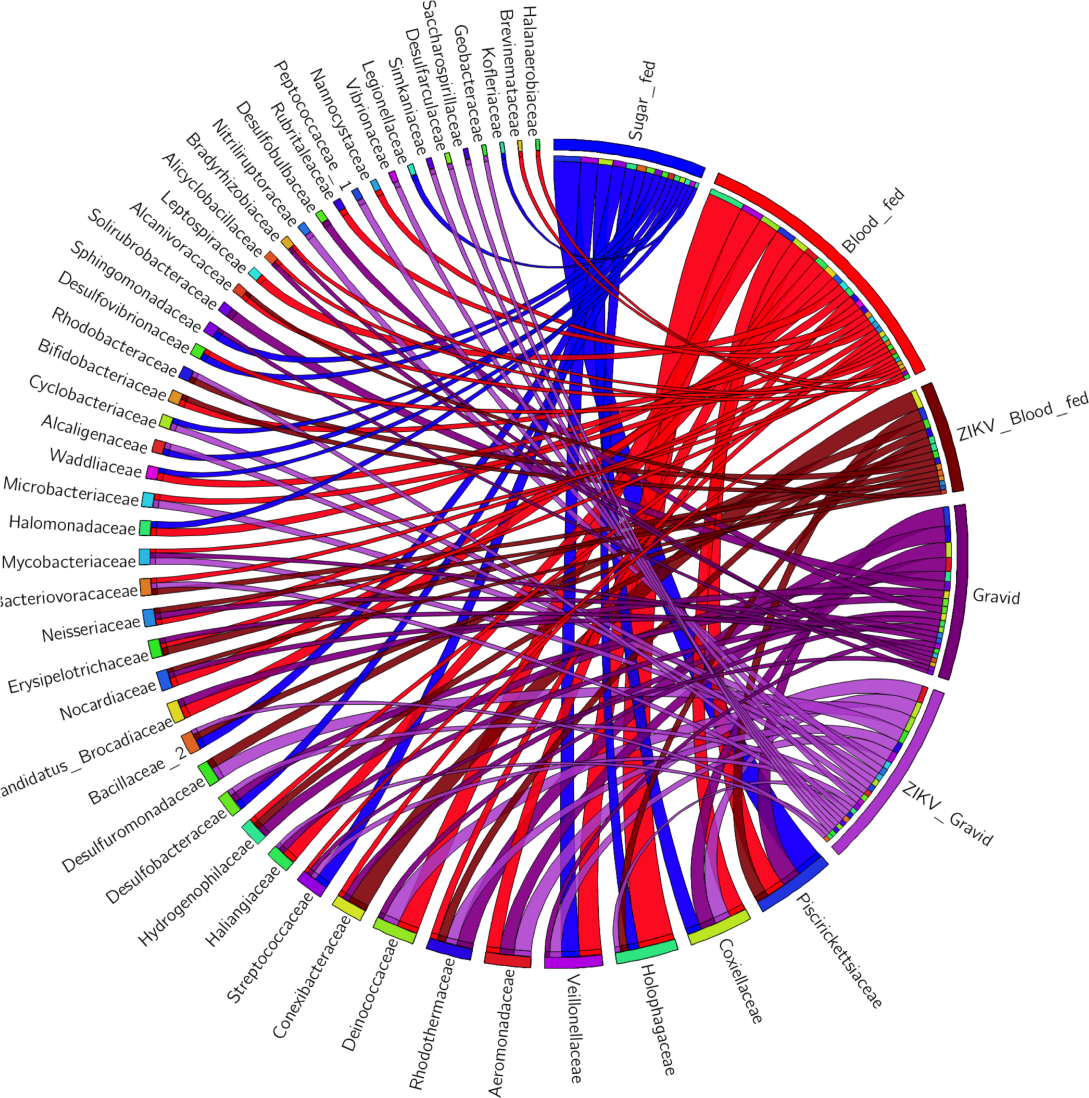
Exclusive bacterial components associated with the *Ae*. *aegypti* group. The circular plot depicts the relative abundance of the exclusive bacterial taxa that were identified per experimental *Ae*. *aegypti* group: sugar-fed (blue), blood-fed (red), ZIKV-infected blood-fed (maroon), Gravid (purple), and ZIKV-infected gravid (lilac). The names of the bacterial f-OTUs and experimental groups are indicated next to their ribbons on the perimeter of the circle. The width of the ribbon and the position of the f-OTUs from the bottom to the top (on each segment) reflect the abundance of each bacterial taxon and their presence in each group.

When the inclusion or exclusion patterns of the bacterial OTUs within the studied experimental *Ae*. *aegypti* groups were considered, subsets of f-OTUs that were present in no more than 4 of the 5 groups were identified in the circular plot shown in Fig 3. Interestingly, 49% of the families that were identified in the whole dataset were absent (not detected by NGS) in at least one test group,34 were Gram-negative and 14 Gram-positive families. When complete exclusion was considered, a total of 14 f-OTUs were identified solely in one experimental group. The group with a higher number of exclusive taxa was the ZIKV-infected gravid group (7), which was interesting since only one f-OTU (*Desulfobulbaceae*) was exclusive to the noninfected gravid females.

**Fig 4:**
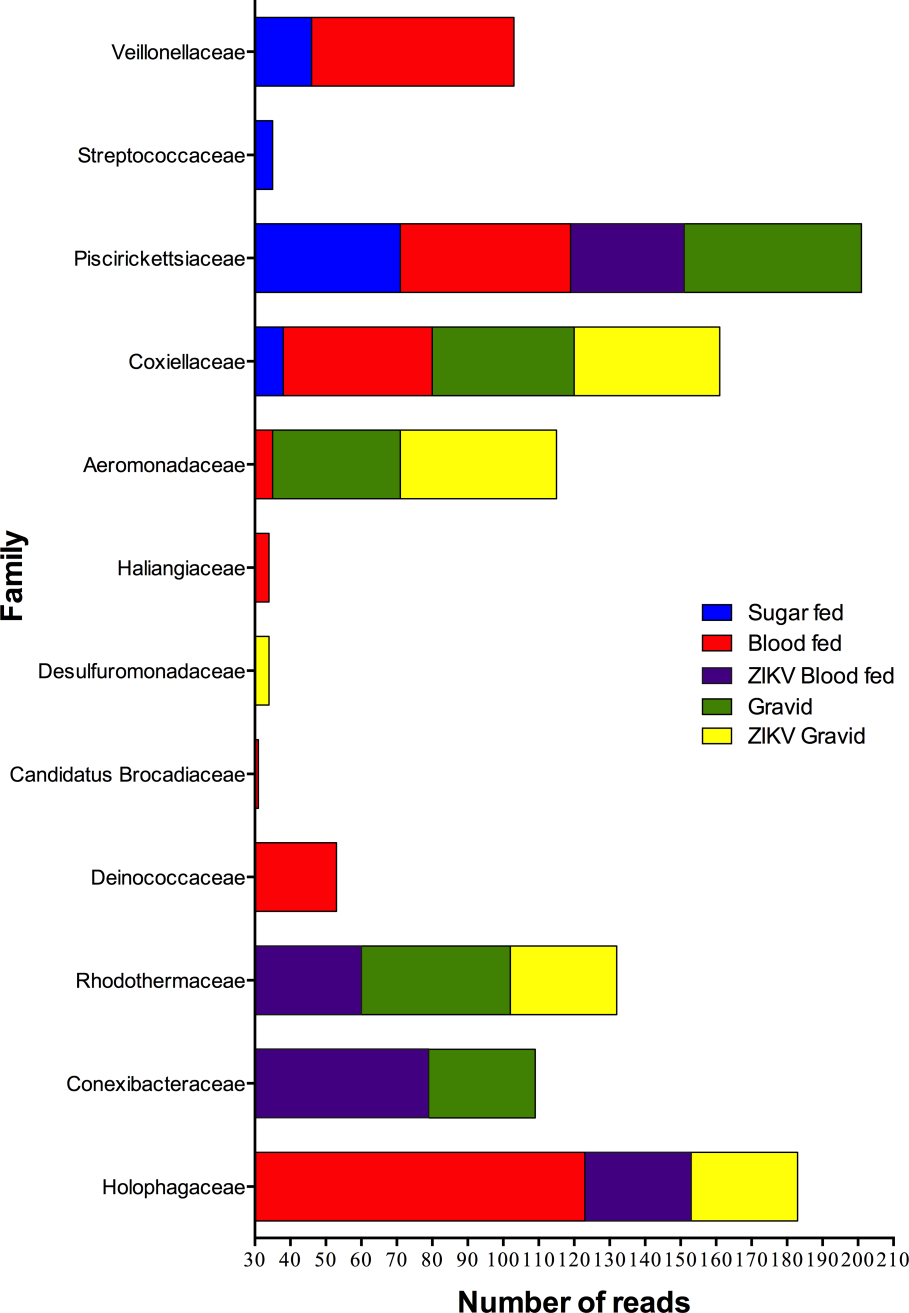
Raw abundance of the exclusive bacterial taxa present in the studied groups of adult mosquitoes. The raw abundance of the arbitrarily filtered f-OTUs that appeared to be present exclusively in the studied groups is shown as stacked bars revealing how community diversity is altered in the experimental groups. Each condition is represented by a color as indicated.

As addressed previously, to further describe fluctuations in community abundance across experimental conditions, the f-OTUs that represented less than 1% of the valid reads (>30 reads) within the experimental *Ae*. *aegypti* groups were arbitrarily excluded, and consequently, 12 taxa were retained (Fig 4).

The exclusive taxa within the communities were low in abundance. This tendency of communities towards low abundance has been described in the human microbiome, wherein niche-specific OTUs with low abundance play key ecological roles by providing essential metabolic benefits to the community network and the host under particular conditions [52]. Following the criteria that were applied, non-core f-OTUs were more prevalent in the blood-fed group. The effects of the reduction in the richness of OTUs in the infected groups were evident when both blood-fed groups were compared.

### CCA analysis of β-diversity, constrained by explanatory biological variables

The native microbiota of insects has been linked to multiple physiological roles, such as digestion, development, and reproduction [3–5,8,15]. Many of the intricate animal-microbe associations are open systems. As a consequence, the host microbiota are continually exposed to external microorganisms, and influenced by environmental factors [53,54]. When hematophagous arthropod vectors are considered, the presence of allochthonous pathogens that are acquired upon blood ingestion may result in the disruption of homeostasis within the autochthonous microbiota [55]. This may be explored by analyzing the structure of the bacterial community in the whole-insect niche using microbial ecological methods. Herein, this question was addressed using a “holobiont” and an insect “macrobe” biological unit [45] by thoroughly considering the open-system nature of animal-microbe consortia [54,56]. In such a scenario, shifts in the signature profiles of bacterial f-OTUs could represent physiological conditions or reflect the impact of pathogen invasion on the multiple compartments that harbor bacteria in mosquitoes [8,15,47,49,57,58].

**Fig 5:**
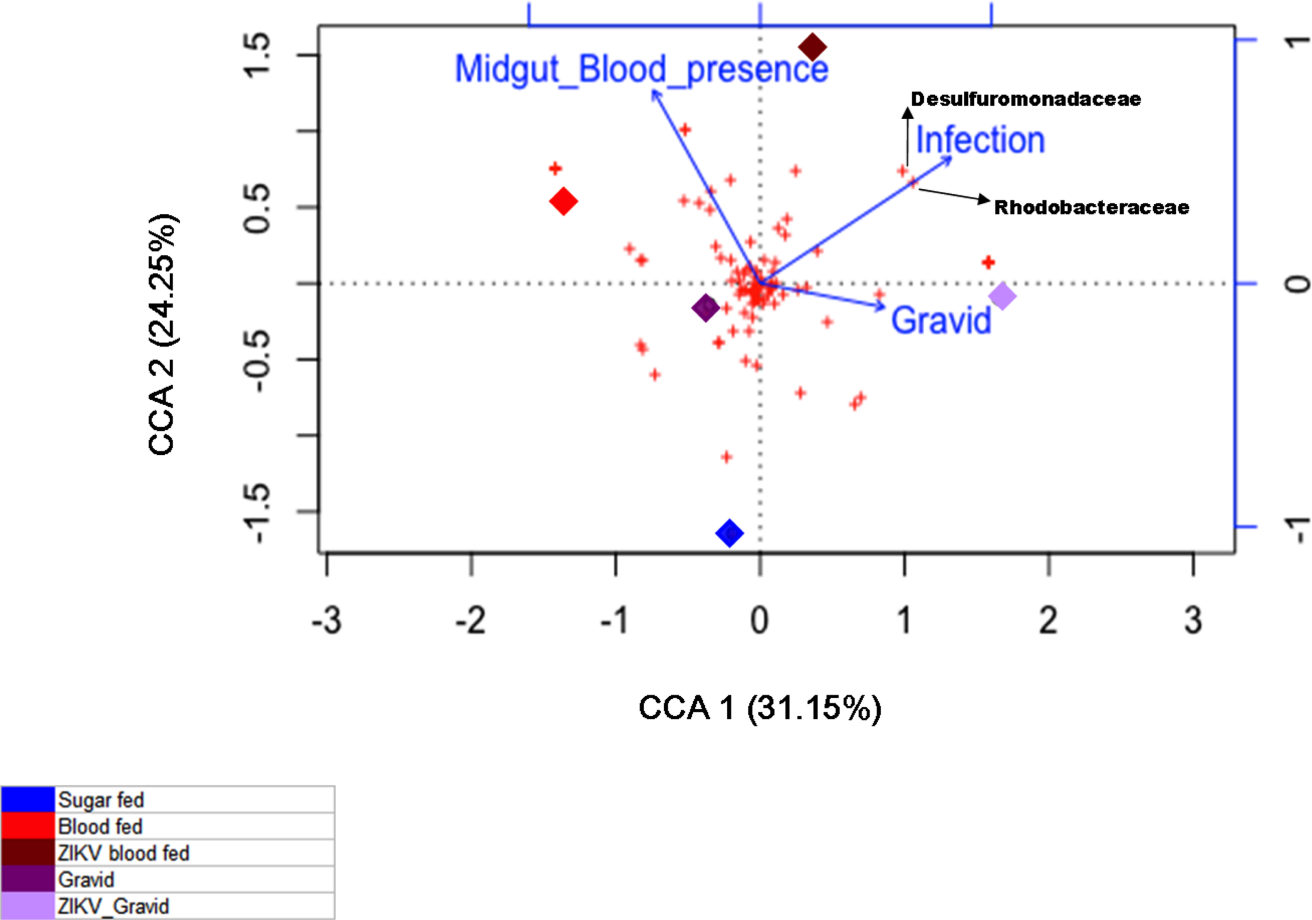
CCA triplot with explanatory biological effectors. The distribution of the experimental groups along the axes, and their orientation regarding the arrow vectors suggest that exposure to blood (red and maroon) and ZIKV infection (maroon and lilac) had an impact on the signature profiles of the bacterial community. The CCA plot, along its two axes (CCA1 = 31.15% and CCA2 = 24.25%), could explain 55.40% of the matrix variance. Experimental groups: sugar-fed (blue), blood-fed (red), ZIKV-infected blood-fed (maroon), Gravid (violet), and ZIKV-infected gravid (lilac).

To address how ZIKV invasion may disturb the ecological balance of the microbiota of *Ae*. *aegypti*, a CCA analysis coupled with an explanatory matrix was performed to support the biological interpretation of shifts in bacterial signatures. First an estimated 55.40% of the variance within the built matrix was contained and explained along the two CCA axes (CCA1 = 31.15% and CCA2 = 24.25%; Fig 5).

The effects that the blood-feeding, infection process, and gravid conditions had on the distribution of the experimental *Ae*. *aegypti* groups and the f-OTUs can be visualized (and interpreted) as the length of each arrow vector shown (Fig 5), and the position that the objects and response variables have are related to them. The bacterial signature profile of the sugar-fed group distinguished this group from all the other groups of mosquitoes that fed on blood, localized in the lower left quadrant of the ordinate space. As this method is based on taxa-environment relationships [30,32] it enabled us to determine the OTUs that responded and thrived under particular environmental variables. This property has been highlighted in diverse studies wherein this and other microbial ecological methods guided the identification of potential indicator species [30,31] that aided the understanding of the bacterial signatures of host organisms [59]. As observed in the CCA triplot, two f-OTUs appeared as potential biomarkers of ZIKV infection: *Rhodobacteraceae* and *Desulfuromonadaceae*. Their abundance reached the maximum value along the explanatory vector that indicated the presence of the ZIKV virus (according to a right-angled projection of the specified crosses onto the “Infection” arrow). Coincidentally, both f-OTUs were exclusively present in the ZIKV- infected blood-fed and ZIKV- infected gravid groups (Fig 3). The distribution of the ZIKV-infected groups in the ordinate space suggested an altered signature profile compared with that of their ZIKV-free counterparts, as indicated by their separated positions along the CCA1 axes, and in reference to the explanatory vectors “Gravid” and “Infection.” Finally, it was unusual to observe the near-180° angle between both gravid groups. This would imply a negative correlation between the two groups based on their signature profiles. Whether the effects of this ecological disturbance on the microbiota of the gravid *Ae*. *aegypti* translated into measurable effects in the microbiota of their offspring microbiota is yet unknown.

### Conclusions

The broad metagenomic survey presented here revealed a dynamic bacterial community that was associated with *Ae*. *aegypti* with signature profiles for each tested physiological condition, and particularly modulated by the presence of ZIKV. The results highlight the existence of a core group of bacterial f-OTUs that remained associated with the mosquito across the tested conditions. We believe the provided bacterial index will enable the research community to perform comparative studies that explore how bacterial symbionts may act as biomarkers of the physiological states of their hosts, and how they respond as a community when arboviruses invade their host. However, the microbiota described in our experimental groups may reflect the fact that these were insects from a colony as well as the tested conditions of the mosquitoes. Basic knowledge of local hematophagous vectors and their associated microbiota is required, as it is relevant to addressing transmission of vector-borne infectious disease in their regional surroundings.

## Supporting information

S1 Fig f-OTU rank bacterial communities associated with the different experimental *Ae*. *aegypti* groups. The circular plot shows the bacterial f-OTU members of the community that were associated with the tested *Ae*. *aegypti* groups: sugar-fed (blue), blood-fed (red), ZIKV-infected blood-fed (maroon), Gravid (purple), and ZIKV-infected gravid (lilac). The names of the bacterial taxa and experimental groups are indicated next to their respective ribbons along the perimeter of the circle. The width of the ribbon and the position of the f-OTUs from the bottom to the top reflect the abundance of the bacterial taxa and their presence within each experimental group.
(TIFF)

S1 Table Data matrix with f-OTU abundance profiles for each tested group. Bacterial community composition at the family taxonomic rank for each tested group.
(PDF)
