# Supplementary figures and images for: Zika virus infection modulates the bacterial diversity associated with Aedes aegypti as revealed by metagenomic analysis
Source: PLoS One. 2018 Jan 2;13(1):e0190352. doi: 10.1371/journal.pone.0190352 (PMC5749803; doi:10.1371/journal.pone.0190352)

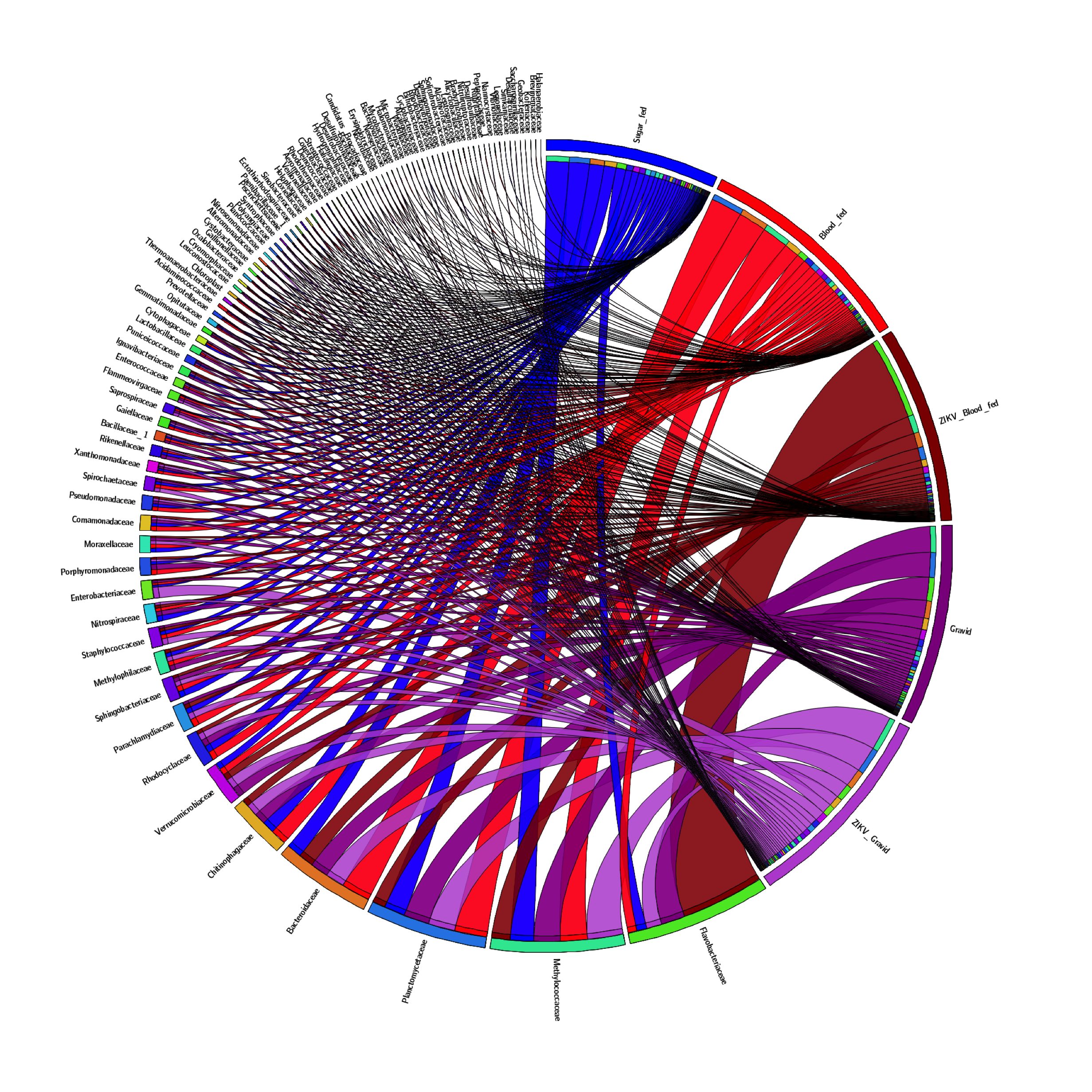

Supplement: S1 Fig — The circular plot shows the bacterial f-OTU members of the community that were associated with the tested Ae. aegypti groups: sugar-fed (blue), blood-fed (red), ZIKV-infected blood-fed (maroon), Gravid (purple), and ZIKV-infected gravid (lilac). The names of the bacterial taxa and experimental groups are indicated next to their respective ribbons along the perimeter of the circle. The width of the ribbon and the position of the f-OTUs from the bottom to the top reflect the abundance of the bacterial taxa and their presence within each experimental group. (TIFF) [file pone.0190352.s001.tiff]
