# Supplementary material for: Zika virus infection modulates the bacterial diversity associated with Aedes aegypti as revealed by metagenomic analysis
Source: PLoS One. 2018 Jan 2;13(1):e0190352. doi: 10.1371/journal.pone.0190352 (PMC5749803; doi:10.1371/journal.pone.0190352)
Supplement: S1 Table — Bacterial community composition at the family taxonomic rank for each tested group. (PDF) [file pone.0190352.s002.pdf]

fOTU	Sugar fed	Blood fed	ZIKV Blood fe	Gravid	ZIKV Gravid
Micrococcaceae	319	370	203	281	266
Gaiellaceae	273	328	145	191	212
Porphyromonadaceae	380	639	233	626	261
Prevotellaceae	122	138	30	114	30
Rikenellaceae	357	241	74	413	216
Bacteroidaceae	1.863	3.563	1.867	2.214	2.346
Cytophagaceae	247	192	58	137	102
Flammeovirgaceae	138	229	115	248	321
Cryomorphaceae	60	102	77	28	37
Flavobacteriaceae	1.259	1.121	10.301	2.946	1.888
Saprospiraceae	170	237	68	299	343
Chitinophagaceae	1.526	1.959	915	1.443	1.420
Sphingobacteriaceae	774	696	341	484	573
Parachlamydiaceae	630	966	537	387	778
Gemmatimonadaceae	85	153	75	177	109
Planctomycetaceae	2.583	4.012	1.590	3.149	3.306
Comamonadaceae	408	580	243	387	266
Oxalobacteraceae	59	97	54	68	18
Gallionellaceae	36	102	35	66	45
Methylophilaceae	480	592	470	625	546
Nitrosomonadaceae	12	36	42	11	154
Rhodocyclaceae	871	1.055	477	870	812
Cystobacteraceae	78	69	26	54	48
Polyangiaceae	51	23	42	19	91
Syntrophaceae	43	49	12	15	97
Enterobacteriaceae	289	281	152	247	1.298
Alteromonadaceae	70	53	52	22	65
Ectothiorhodospiraceae	23	72	23	16	38
Methylococcaceae	2.910	3.350	2.279	3.274	4.393
Moraxellaceae	528	485	233	362	446
Pseudomonadaceae	409	479	221	331	294
Sinobacteraceae	39	63	27	19	20
Xanthomonadaceae	270	369	155	270	263
Spirochaetaceae	372	412	126	291	476
Opitutaceae	121	168	74	153	82
Puniceicoccaceae	167	202	125	163	192
Verrucomicrobiaceae	792	804	827	1.273	1.237
Chloroplast	100	130	39	44	47
Bacillaceae_1	252	267	150	303	190
Paenibacillaceae _1	22	62	53	51	10
Planococcaceae	67	50	18	29	84
Staphylococcaceae	450	491	359	318	750
Enterococcaceae	145	217	125	323	193
Lactobacillaceae	116	201	69	230	154
Leuconostocaceae	26	39	72	124	63

Thermoanaerobacteracea	73	76	51	104	62
Acidaminococcaceae	42	136	60	136	41
Ignavibacteriaceae	237	151	117	262	182
Nitrospiraceae	654	407	355	557	303
Holophagaceae	28	93	20	0	14
Microbacteriaceae	0	16	0	0	20
Mycobacteriaceae	0	10	0	12	17
Nocardiaceae	0	24	11	14	0
Bifidobacteriaceae	0	17	13	0	0
Nitriliruptoraceae	0	0	0	0	19
Conexibacteraceae	0	14	49	18	0
Solirubrobacteraceae	0	0	0	25	0
Cyclobacteriaceae	14	0	0	0	17
Rhodothermaceae	0	20	22	42	29
Simkaniaceae	0	0	0	0	13
Waddliaceae	13	19	0	0	0
Deinococcaceae	0	53	0	15	40
Candidatus Brocadiaceae	0	31	0	20	0
Bradyrhizobiaceae	0	10	0	11	0
Rhodobacteraceae	0	0	16	0	14
Sphingomonadaceae	19	0	0	10	0
Alcaligenaceae	16	0	0	0	16
Hydrogenophilaceae	0	14	19	26	0
Neisseriaceae	0	15	13	15	0
Bacteriovoracaceae	0	13	16	0	14
Desulfarculaceae	0	0	0	0	13
Desulfobacteraceae	23	0	0	20	15
Desulfobulbaceae	0	0	0	19	0
Desulfovibrionaceae	17	13	0	0	0
Desulfuromonadaceae	0	0	24	0	34
Geobacteraceae	0	0	0	0	11
Haliangiaceae	0	34	0	17	11
Kofleriaceae	10	0	0	0	0
Nannocystaceae	0	16	0	0	0
Vibrionaceae	0	0	0	0	15
Aeromonadaceae	0	35	0	36	44
Coxiellaceae	38	42	0	40	41
Legionellaceae	14	0	0	0	0
Saccharospirillaceae	0	0	0	0	12
Alcanivoracaceae	0	13	10	0	0
Halomonadaceae	16	21	0	0	0
Piscirickettsiaceae	71	48	32	50	0
Brevinemataceae	0	10	0	0	0
Leptospiraceae	0	22	0	0	0
Rubritaleaceae	0	18	0	0	0
Alicyclobacillaceae	0	10	0	12	0

Bacillaceae_2	25	0	16	0	11
Streptococcaceae	35	10	0	11	15
Peptococcaceae_1	0	0	0	0	17
Halanaerobiaceae	0	10	0	0	0
Erysipelotrichaceae	0	13	19	13	0
Veillonellaceae	46	57	0	14	29
# of reads	21.383	27.235	24.072	24.594	25.649
